# An Overview of New Insights into the Benefits of the Seagrass *Posidonia oceanica* for Human Health

**DOI:** 10.3390/md19090476

**Published:** 2021-08-25

**Authors:** Marzia Vasarri, Anna Maria De Biasi, Emanuela Barletta, Carlo Pretti, Donatella Degl’Innocenti

**Affiliations:** 1 Department of Experimental and Clinical Biomedical Sciences, University of Florence, Viale Morgagni 50, 50134 Florence, Italy; marzia.vasarri@unifi.it (M.V.); emanuela.barletta@unifi.it (E.B.); 2 Interuniversity Center of Marine Biology and Applied Ecology “G. Bacci” (CIBM), Viale N. Sauro 4, 57128 Livorno, Italy; debiasi@cibm.it (A.M.D.B.); pretti@cibm.it (C.P.); 3 Department of Veterinary Sciences, University of Pisa, Viale delle Piagge 2, 56124 Pisa, Italy

**Keywords:** *P. oceanica*, angiosperm, marine natural products, seagrass, secondary metabolites, phytochemicals

## Abstract

*Posidonia oceanica* (L.) Delile is a Mediterranean-endemic angiosperm often described for its great ecological importance. Despite evidence of a millennia-old relationship between *P. oceanica* and humans, as well as traditional medicine applications, the potential benefits of *P. oceanica* for human health have been documented only recently. This review aims to compile newly acquired knowledge on *P. oceanica* bioactive properties that allow the scientific community to look at this plant as a promising source of natural therapeutical products for human health. Experimental investigations conducted in both in vitro cellular-based and in vivo animal models pave the way for new research projects aiming at the development of alternative and complementary therapeutic strategies based on *P. oceanica* against a wide range of pathological conditions.

## 1. Introduction

Seagrasses are flowering and rhizomatous plants that grow only in marine environments and form extensive underwater meadows. They are categorized into four families in the monocotyledonous order Alismatales: Cymodoceaceae, Hydrocharitaceae, Posidoniaceae, and Zosteraceae. They are among the most valuable coastal ecosystems on the planet in terms of the goods and services they provide [1,2].

Although often confused with algae, seagrasses are marine vascular plants deriving from higher terrestrial plants that have secondarily colonized marine habitats. Hence, they generally share most of their primary and secondary metabolism features with their relatives of the Alismatales order living in terrestrial and freshwater habitats [3].

Five species of seagrasses populate the Mediterranean Sea, *Posidonia oceanica* (L.) Delile, *Cymodocea nodosa* (Ucria), *Zostera marina* L., *Zostera noltii* Hornemann, and *Halophila stipulacea* (Forsskål) Ascherson (non-indigenous species) [4].

*P. oceanica*, a member of the Posidoniaceae family, is the most important species of seagrass for the Mediterranean Sea’s ecological coastal balance due to its widespread distribution and abundance [5].

*P. oceanica* is a slow-growing marine phanerogam (>10 cm year^−1^), organized in roots, rhizomes, and leaves [5,6].

Under favorable environmental conditions, *P. oceanica* colonizes vast underwater areas, forming extensive meadows from the surface to 40 m depth (although it has been recorded in particularly clear waters until 48 m depth), covering about 1.5% of the total Mediterranean Sea surface [7,8,9].

*P. oceanica* meadows are a highly complex and well-structured ecosystem known for their high biodiversity and great primary productivity, which is of paramount importance for the health of the Mediterranean coasts [10,11].

For this reason, in the last twenty years, *P. oceanica* has become one of the main targets of the protection and management of the Mediterranean marine environment. It has been included by the European Union’s Habitat Directive (92/43/CEE) among the habitats of priority interest [7,12,13] and it is protected under the Bern and the Barcelona Conventions, and other legislation at a national level. Moreover, the MFSD (2008/56/EC) selected *P. oceanica* as representative species of the angiosperm quality elements for the Mediterranean marine environment [14].

*P. oceanica*, in addition to its inestimable ecological value for the Mediterranean marine environment, has been used in the past for other purposes in the construction, industrial, and commercial sectors, as well as in the pharmacological field. Besides the knowledge handed down over time, in the last few years, a growing body of evidence have reinforced the awareness of the beneficial properties of *P. oceanica* phytocomplex for human health [15,16,17,18,19,20], thus enhancing the scientific community interest in *P. oceanica* as an unexplored resource of phytotherapeutic compounds.

This overview for the first time focuses on recent advances in determinations of *P. oceanica* biological activities, in particular its potential health benefits.

## 2. Ecological Significance of *P. oceanica* Seagrass Ecosystem

**Figure 1:**
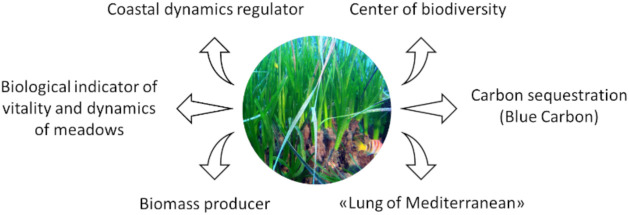
Summary list of the ecological significance of *P. oceanica* seagrass.

In the Mediterranean Sea, the dominant endemic seagrass *P. oceanica* forms widespread ecosystems with high levels of biodiversity and productivity [7,21,22]. Figure 1 summarized a variety of ecologically and geologically important functions of *P. oceanica* [23,24,25].

*P. oceanica* is considered of paramount importance being a hot spot of biodiversity, hosting about 20–25% of all species living in the Mediterranean Sea. Leaves and rhizomes offer substrata suitable for settlement and growth of a number of sessile organisms that form stratified assemblage guaranteeing the development of microniches for different taxonomic groups. About one-hectare meadows can host up to 350 different species of animals, residents, or migrants, thus offering shelter and nourishment to cephalopods, bivalves, gastropods, echinoderms, tunicates, and fish, also of considerable economic importance. The rich and diversified animal and plant community populating these grasslands contributes to create a complex trophic network, highly efficient and productive, capable of exporting energy to other systems [7,26]. Other studies have pointed out that the leaves of *P. oceanica* are a rich source of biochemical components, major and essential trace elements, making the seagrass of great nutritional value to marine organisms and an essential role in the marine food chain [27].

*P. oceanica* seagrass produces a large amount of plant biomass, ranging between 400 and 2500 gDW/m^2^/y^−1^, with decreasing values as depth increases. The contribution of the autotrophic epiphytes commonly associated with this plant raises these values dramatically to 2000–3000 gDW/m^2^/y^−1^. Because of these characteristics, *P. oceanica* meadows are regarded as one of the most productive of the Earth’s ecosystems [7,26].

*P. oceanica* is known as “the lung of the Mediterranean” because it can emit up to 20 L of oxygen per day per square meter of meadows, making it the most important source of oxygen supplied by coastal waters [24,28].

*P. oceanica* ecosystem is also important in coastal dynamics, as it contrasts gradual silting, stabilizes sediment, protects the coastline from erosion through seabed consolidation, and attenuates swells and waves through the oscillatory movement of its leaves [29,30].

The floating dead leaves of *P. oceanica* (massive in autumn) that reach the coast create dense deposits along with other debris and deposits; when conditions allow, they can consolidate and produce a very compact and strong structure called “banquettes”, which are essential for protecting the coast from erosion [7,31,32].

Coastal areas are often characterized by environmental disturbances both due to natural events and anthropological activities (overexploitation, physical modification, nutrient and sediment pollution, introduction of non-native species, climate change), with consequent impacts on the marine ecosystem [33].

Seagrass meadows are subjected to all the mentioned threats so that they are presently experiencing a decline globally [34] and are among the most threatened ecosystems on earth [35]. This is especially true for the Mediterranean, a semi-enclosed basin under severe demographic, urban, and industrial pressures, and where climate change is having a significant impact [36]. Global acidification has been clearly demonstrated to be a new and concerning threat to the health of marine and terrestrial ecosystems. As a result, several studies have been conducted to investigate the role of coastal vegetation in carbon sequestration (Blue Carbon). *P. oceanica* has the most organic carbon stores that have been documented [37] and can be considered an “outlier” within seagrass species. Under this contest, the loss of *P. oceanica* meadows assumes greater importance.

Its regression may result in the erosion and rapid remineralization of the carbon-rich soils stored beneath the canopy, in particular in exposed locations, thus releasing CO_2_. Furthermore, in areas where *P. oceanica* dies and the leaf canopy disappears, the underlying *matte* is no longer protected against erosion; dismantling of the matte will amplify the remineralization process accelerating climate change effects in a similar way to fossil fuels.

For this reason, *P. oceanica* has long been used as an excellent biological indicator of the vitality and dynamics of meadows and human influence on the marine environment. [24,25,38,39].

Hence, *P. oceanica* is an essential resource for humans, but at the same time, it represents a fragile ecosystem with recovery times in hundreds of years. Faced with the dramatic threat of *P. oceanica* meadows regression, in the last few years, numerous environmental projects have been carried out for transplanting and reforesting the seagrass in the seabed to support the conservation of *P. oceanica* as one of the most precious and important habitats for marine environment and humans.

In this context, a recent study has developed an efficient system for the regeneration of *P. oceanica* seagrass by storing free cells at low temperatures and initiating cell encapsulation. Cell encapsulation was the only successful method to acclimatize cells to salinity, preserve artificial material for sowing, and obtain embryos. This system could help solve problems related to the intractable nature of in vitro regeneration of marine phanerogams [40].

## 3. Historical and Traditional Medical Uses of *P. oceanica*

Even before discovering its enormous ecological value, archaeological and historical evidence tells of a relationship lasting for millennia between humans and *P. oceanica* seagrass.

Curiously, in the cave of Lazaret, in the French Maritime Alps, remains of leaves were found dating back to the end of the Riss glaciation (over 100 thousand years ago), presumably used as a bed by the occupants [41].

**Figure 2:**
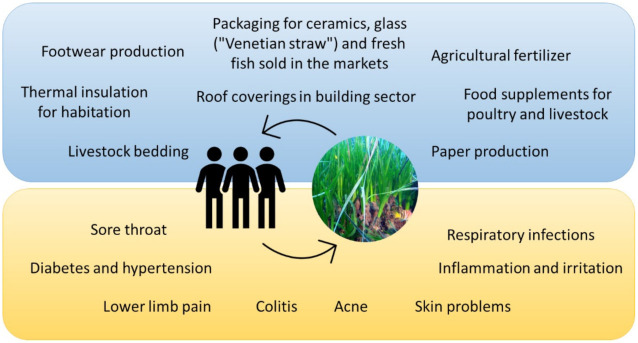
Schematic list of traditional roles of *P. oceanica* seagrass in both the commercial, industrial, and agricultural sectors (blue box) and in the medical field for the treatment of human health disorders (yellow box).

*P. oceanica* claims numerous traditional uses in human history in various commercial and industrial sectors thanks to its innumerable properties (Figure 2). The first details on *P. oceanica* use in human civilization date back to ancient Egypt, where the *P. oceanica* egagropils (agglomerates of fairly spherical fibers from dead leaves of *P. oceanica* formed by hydrodynamics in shallow waters and then rejected on the beaches) were used for the production of footwear [42,43].

Moreover, for centuries *P. oceanica* leaves were used by the Venetians to wrap and transport their famous delicate glass, these leaves were in fact known as “Venetian straw” [44]. They were also used as packaging for ceramics and even for fresh fish sold in the markets. Furthermore, at the beginning of the twentieth century, the coastal populations of northern Africa (Egypt, Libya, Tunisia) exploited the biological characteristics of *P. oceanica* also in the building sector by using dry leaves as roof coverings [45] and thermal insulation for habitation [44]. *P. oceanica* also claims use in the agricultural field. Traditionally, *P. oceanica* leaves were used as agricultural fertilizer to improve the characteristics of the soil, a use which went for a long time by the farmer of the Mediterranean coast [46,47]. However, in 2009, the biochemical and mineral content of *P. oceanica* was more thoroughly studied. The *P. oceanica* mineral content, especially calcium (Ca, 3890.00 mg/100 g), phosphorus (P, 930.00 mg/100 g), and sodium (Na, 2765.00 mg/100 g), was found to be significantly higher than the allowable concentrations proposed in Compost Management Program (2012) (Ca: 3000, P: 250 and Na: 1000 mg/100 g) [27,48], thus revising the traditional use of *P. oceanica* as an organic fertilizer and/or compost.

In Tunisia, *P. oceanica* leaves were used as livestock bedding, still in use today thanks to their antifungal and insect repellent properties. Considering the nutritional value of *P. oceanica* leaves similar to that of fodder plants, they were also used as food supplements for poultry and livestock [7]. Among other uses, *P. oceanica* was employed in the production of paper in the late nineteenth century [49,50].

The material extracted from *P. oceanica* has also been used experimentally for other industrial applications, such as for methane and nitrocellulose production [51]. Beyond varied applications in human activities, *P. oceanica* seagrass has traditionally been used also as a medical plant in the treatment of various human disorders (Figure 2). The first information on the *P. oceanica* healing properties comes from ancient Egypt, where it was supposedly used for sore throat and skin problems [52]. An old botany handbook of Cazzuola also mentions *P. oceanica* as a popular pharmacopeia product [53]. It has also been documented that *P. oceanica* leaves were used to treat inflammation and irritation, but also as a remedy for acne, lower limb pain, and colitis [54]. On the other hand, the curious use of *P. oceanica* as padding for cushions and mattresses dates back to the sixteenth century. It is believed that this practice served to prevent respiratory infections and alleviate the conditions of people with tuberculosis. The use of *P. oceanica* leaves decoction as a natural remedy for diabetes and hypertension by the inhabitants of the coastal areas of Western Anatolia belongs to a more recent tradition [55].

Queen of the seas for 120 million years, *P. oceanica* has therefore proved to be a precious ally for the care of human health.

## 4. Phytochemical Compounds of *P. oceanica* Leaves

Numerous characterization studies have shown that *P. oceanica* is a rich source of secondary metabolites (Table 1), mainly represented by phenolic compounds [56], essential in plant self-protection from photosynthetic stress, reactive oxygen, anthropogenic pressures, predators, and pathogens [57,58].

Chicoric acid is reported as the major constituent of *P. oceanica* leaves [15,59,60]. However, *P. oceanica* leaves also abound with caftaric, gentisic, chlorogenic, caffeic, ferulic, cinnamic, gallic, and *p*-coumaric acids [15,19,56,59,61]. In addition, quercetin, myricetin, kaempferol, and isorhamnetin are found in the diethyl layer of a hydrochloric acid solution used for extraction from *P. oceanica* leaves, were the most represented secondary metabolites belonging to the class of flavonoids [62,63].

Other phenolic derivatives were identified and quantified in *P. oceanica* leaves extracts, including phloroglucinol, pyrocatechol, pyrogallol, vanillin aldehyde, 4-hydroxybenzaldehyde, 3,4-dihydroxybenzaldehyde, benzoic acid, *p*-hydroxybenzoic acid, *p*-anisic acid, vanillic acid, syringic acid, proanthocyanidins, and calchones (as phloretin and phloridzin) [56,59,62,63].

The presence of long-chain fatty acids in *P. oceanica* seagrass is another distinguishing feature. The lipid fraction of *P. oceanica* leaves extracts was discovered to be primarily composed of palmitic, palmitoleic, oleic, and linoleic acids, as well as the phytosteroids campesterol, stigmasterol, and β-sitosterol [64]. Polyphenol-derived lignin was discovered to be present in all plant tissues [65].

In 2013, posidozinol, new sesquiterpene alcohol, was isolated from a chloroform extract of *P. oceanica* leaves [66].

Scientific research in recent years has shed light on the ability of the *P. oceanica* secondary metabolites to exert biological properties effectively due to the synergistic action of its phytochemical components. The most recent developments in the field of herbal medicine on the health-promoting properties and benefits that *P. oceanica* could provide are listed and summarized below

## 5. *P. oceanica* Bioactive Properties: From Tradition to New Horizons

### 5.1. Antibacterial, Antifungal, and Antiviral Role

As early as 1989, Bernard and Pesando attributed to an extract of *P. oceanica* rhizomes for the Mediterranean maritime flora anti-bacteria and anti-fungal properties [67]. Subsequent studies showed that extracts from *P. oceanica* leaves also had antibacterial activities against both Gram-positives and Gram-negatives, with particular efficacy against *P. aeruginosa* and *S. aureus* [68]. *P. oceanica* has also shown antiviral capabilities, notably, in 2018 Farid et al. obtained that *P. oceanica* ball extracts inhibited H5N1 virus infection by 45% [69].

In recent years, scientific research has shown increasing interest in identifying natural compounds with antioxidant and antimicrobial activity with potential applications in food science [70]. The current trend in food processing is focusing on the use of natural compounds, which are considered safe alternatives to chemical additives [71,72]. The exploration of new phytocompounds with these functional properties is a topic of great current interest with human well-being as the main goal. In fact, some microorganisms can have a negative impact on food quality, safety, and shelf life, and oxidative processes can have an impact on food quality, affecting human health.

*P. oceanica* seagrass, as an antimicrobial and antioxidant agent, has piqued the scientific community’s interest in its potential applications in food science. A number of studies have looked into the potential applications of *P. oceanica* in the field of food preservation by reducing and eliminating pathogens and spoilage microorganisms in food [73,74].

In 2017, a *P. oceanica* leaves extract was found to reduce microbial spoilage in fresh peaches, particularly that caused by the Pseudomonas population, and so proposed for use on fresh-cut fruit [73]. This discovery could pave the way for the use of novel natural extracts on ready-to-eat vegetables, increasing the consumer appeal of these healthy foods.

The growing interest in *P. oceanica* extracts in the field of food science has prompted researchers to develop more environmentally friendly alternative methods that avoid the use of toxic solvents to optimize the processes of extracting phytochemical compounds from *P. oceanica* leaves. Indeed, the use of a water-soluble extraction method has been shown to more ecologically ensure the maintenance of *P. oceanica* biological properties, including antioxidant, antifungal activities, especially against food-borne fungi—i.e., *P. digitatum*, *P. italicum*, *P. expansum*, *B. cinerea*, *G. candidum*, and *A. niger*—and antiviral against feline calicivirus and murine norovirus. Future investigations aim to maximize extraction yields and antioxidant, antifungal, and antiviral properties of the extracts [74].

### 5.2. Insights into the Bioactivities of P. oceanica with Potential Human Health Applications

Comprehending the potential of the natural world to produce secondary metabolites is remarkable in a wide range of fields, including drug discovery. With more than 70% of the Earth’s surface, the marine environment is the largest terrestrial habitat and prolific supplier of biologically active compounds because of its extraordinary biodiversity, which is relatively unexplored compared to terrestrial environments.

The variety of bioactive marine compounds with great pharmaceutical potential is unique, and their production is aided by the chemical and physical conditions of the sea. Marine natural products with a wide range of biological activities have increasingly attracted the attention of many researchers as a useful resource for developing drugs for the management of human health [75].

Beyond its invaluable ecological and environmental significance, scientific research in recent years has fueled interest in the hitherto unexplored phytochemical properties of *P. oceanica* for potential pharmaceutical applications in human health.

**Figure 3:**
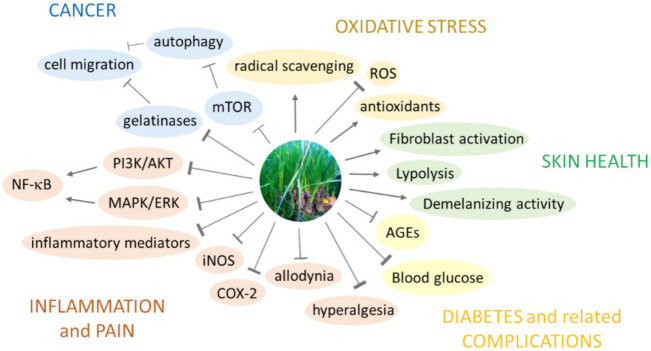
Scheme of the different modes of action of *P. oceanica* and possible therapeutic targets for human health.

Recent studies have shown that *P. oceanica* acts through a variety of mechanisms of action, establishes molecular interactions, and triggers multiple signaling pathways. These insights are schematized in Figure 3 and described in detail below. 

The high content of phenolic compounds and their derivatives make *P. oceanica* leaves a rich source of antioxidant molecules. Physiologically, *P. oceanica* exploits its ability to stimulate the functionality of antioxidant enzymes as a defense mechanism against various environmental pressures [76], including increasingly frequent and strong thermal stress in the face of human-induced climate change [77] or stressful conditions such as competition from invasive macroalgae [76,78], which strongly contribute to the phenomenon of seagrass regression in the Mediterranean.

In scientific research, many natural products with antioxidant properties have found applications in a wide range of fields, including biomedicine. The exploration of new natural products with these functional properties is a topic of great current interest for human well-being as the main focus.

*P. oceanica* seagrass may find application in the prevention of cellular damage and oxidative stress-related diseases, due to its antioxidant power. *P. oceanica* leaves extract, rich in chicoric acid, was shown to have effects on human dermal fibroblast proliferation and collagen production, as well as anti-melanogenic properties in the human melanoma cell line MeWo and lipolytic properties in human subcutaneous preadipocytes; these properties were attributable to its antioxidant power [15]. Therefore, the antioxidant activity of *P. oceanica* extract may be associated with protection against wrinkle formation, skin aging, unwanted hyperpigmentation, and cellulite, in which oxidative stress plays a crucial role.

The imbalance between the production of reactive oxygen species (ROS) and their elimination by protective mechanisms often underlies oxidative damage and thus the onset and progression of inflammatory diseases [79]. Under conditions of inflammation, the uncontrolled production of ROS during the so-called “oxidative burst” is closely related to the histolesivity of inflammation, which represents the “dark” side of the inflammatory process associated with suffering cells and tissues. A *P. oceanica* leaves extract was shown to decisively suppress the expression level of key inflammation-associated enzymes, namely inducible nitric oxide synthase (iNOS) and cyclooxygenase-2 (COX-2), impairing the production of cell- and tissue-damaging metabolites, like nitric oxide (NO) as a precursor of peroxynitrite, and generally the production of intracellular ROS in lipopolysaccharide (LPS)-stimulated murine macrophages RAW264.7 [17]. The transcription factor nuclear factor-B (NF–κB) regulates genes involved in inflammation and tumorigenesis [80]. One possible mechanism by which *P. oceanica* exerts its anti-inflammatory activity is the inhibition of NF–κB activity. Vasarri et al. (2019) found that *P. oceanica* inhibited LPS-induced NF–κB activation by preventing phosphorylation and nuclear translocation of the p65 subunit of NF–κB [17].

In the NF–κB signaling pathway, IκB is an inhibitory protein that binds to inactive cytosolic NF–κB. Following a pro-inflammatory stimulus, IκB kinase (IKK) phosphorylates IκB, degrading it and allowing NF–κB to translocate into the nucleus. *P. oceanica* has been shown to prevent nuclear translocation of NF–κB by restoring basal levels of IκB. The anti-inflammatory activity afforded by *P. oceanica* also occurred by modulating signaling pathways upstream of NF–κB pathway, i.e., mitogen-activated protein kinase (MAPK)/extracellular signal-regulated kinase (ERK) and phosphatidylinositol 3-kinase (PI3K)/protein kinase B (AKT). Notably, *P. oceanica* inhibited LPS-induced activation of ERK and AKT [17].

A further in vivo study on different models of acute inflammatory pain in CD-1 mice provides pharmacological evidence for the anti-inflammatory and analgesic role of a *P. oceanica* leaves extract. Acute oral administration of the extract has been shown to dose-dependently inhibit both inflammation-induced hypersensitivity after intraplantar injection of carrageenan and inflammatory pain induced by intraplantar injection of interleukin–1β (IL–1β) or formalin or against the physiological pain threshold in naive animals [81]. *P. oceanica* also reduced tissue myeloperoxidase (MPO) activity and tissue concentrations of inflammatory cytokines, such as tumor necrosis factor-α (TNF–α) and IL–1β [81].

Other in vitro bioactivity studies have described the ability of a *P. oceanica* leaves extract to inhibit the migration and invasiveness of tumor cells, such as human fibrosarcoma HT1080 cells [19] and human neuroblastoma cells SHSY5Y [18]. This role has been related to its ability to inhibit the expression and activity of metalloproteases 2 and 9 proteolytic enzymes playing a key role in the processes of tumor metastasis by degrading the extracellular matrix [19]. The molecular mechanisms associated with the anti-migratory effects in cancer cells involve the activation of autophagy [16], a conserved evolutionary process that mediates the degradation of cytoplasmic material [82].

Leri et al. (2018) suggested that the activation of autophagy afforded by *P. oceanica* is due to the concomitant and reverse modulation of ERK and AKT signaling pathways culminating in the inhibition of the mammalian target of rapamycin (mTOR), the master regulator of autophagy [16]. The interaction between *P. oceanica* and the ERK and AKT pathways provides possible explanations for many of the beneficial effects of *P. oceanica*.

Typically, the mode of action of anticancer drugs is based primarily on differential toxicity and sensitivity of actively growing tumor cells compared with normal cells. The reported ability of the *P. oceanica* phytocomplex to act against cell migration by a completely nontoxic mechanism makes it a promising reservoir of potent and safe molecules that can defend against neoplasms, but also against other chronic pathophysiological processes, such as neurodegeneration, inflammation, and skin aging, in whose progression gelatinolytic activity is the hallmark [16,19].

Many natural products have demonstrated effective anticancer activities for many years, resulting in valuable chemotherapeutic and cancer prevention agents [83]. However, the reduced bioavailability of natural products, which is often due to their hydrophilic or lipophilic nature, limits their use in anticancer therapy as well as the treatment of other diseases [84].

Different nanosystems (e.g., liposomes, polymer nanocapsules, and dendrimers) are designed to deliver drugs and are composed of materials that can mask the unfavorable biopharmaceutical properties of the encapsulated molecule [84]. The use of these nano-delivery systems can increase the in vivo stability and bioavailability of the delivered natural products, but also improve their selective activity against cancer cells. In this context, *P. oceanica* leaves extract has been found suitable for the use of nanotechnology. Piazzini et al. (2019) demonstrated that the use of polymeric nanomicelles of Soluplus^®^, a tri-block copolymer consisting of polyvinylcaprolactam-polyvinylacetate-polyethylene glycol, to deliver the extract allowed both to increase the aqueous solubility of *P. oceanica* phytocomplex and to improve its bioactivity in terms of inhibition of human neuroblastoma SHSY5Y cell migration. The prolonged release of the extract from the nanomicelles could be the factor responsible for the demonstrated biological enhancement of *P. oceanica* [18]. The development of an appropriate delivery nanosystem of *P. oceanica* may offer an advanced approach to improve the bioavailability and/or optimize the solubility and stability of *P. oceanica* extract promoting its potential health benefits.

Treatment with medicinal plants is as old as mankind. Knowledge of the use of medicinal plants is the result of many years of fighting disease, during which humans learned to look for medicine in nature. Modern science has recognized the active action of medicinal plants and has included a number of drugs of plant origin known to ancient civilizations and used over millennia [85]. The use of *P. oceanica* seaweed as a medicinal plant dates back to Mediterranean civilizations, which used it to treat a variety of human health problems, as described above. *P. oceanica* leaves decoction was used as a natural remedy for diabetes and hypertension by the people of western Anatolia [55]. In this regard, Gokce et al. (2008) demonstrated that oral administration of an extract of *P. oceanica* in alloxan-induced diabetic rats strongly restored the activity of antioxidant enzymes and decreased the process of lipid peroxidation, as well as reduced blood glucose [55]. The ramification between the antioxidant, vasoprotective, and hypoglycemic properties of *P. oceanica* extract could provide therapeutic benefits in diabetes-related endothelial dysfunction and oxidative damage.

Persistent hyperglycemia or uncontrolled diabetes has the potential to cause severe complications; an unavoidable consequence of a long-lasting hyperglycemic state is increased accumulation of advanced glycation end products (AGEs), a heterogeneous group of products obtained from the nonenzymatic Maillard reaction between circulating macromolecules and free-reducing sugars. *P. oceanica* leaves extract has been shown to inhibit the formation of AGEs in vitro [20]. The dual antidiabetic and anti-glycation role of *P. oceanica* may help manage diabetes and prevent associated complications.

**Figure 4:**
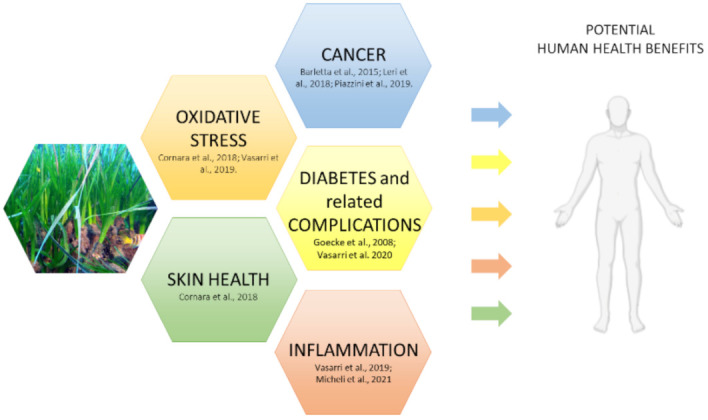
Schematic representation of the potential benefits of *P. oceanica* phytocomplex in human health.

Overall, *P. oceanica* phytocomplex is indicated to the entire scientific community as a reservoir of molecules potentially exploitable in various applications in human health (Figure 4).

## 6. Conclusions

*P. oceanica* is a member of the Posidoniaceae family and the most important species of the Mediterranean angiosperms.

The idea behind this review was to highlight that until recently researchers have described the roles of this angiosperm linked to the sea environmental health, and only indirectly to the health of humans as recipients of good environmental conditions. Despite its ecological role, this overview of the bioactive properties of *P. oceanica* allows the scientific community to consider this plant as a promising resource for human health.

In recent studies, *P. oceanica*—rich in secondary metabolites—has shown some important roles such as antioxidant, anti-inflammatory, antidiabetic, anti-glycation properties, and the ability to suppress cancer cell migration. It has also been shown that *P. oceanica* can act as an activator of autophagy through interaction with specific signal transduction pathways.

A future challenge should include studying the bioavailability of *P. oceanica* secondary metabolites and thus the actual beneficial effects on human health to propose this angiosperm for potential pharmacological applications.

## Figures and Tables

**Table 1:** Structures and molecular formulas of major compounds isolated from *P. oceanica* leaves.

Compound	Molecular Formula	Structure	References
Chicoric Acid	C_22_H_18_O_12_	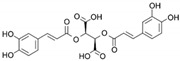	[15,59,60,61]
Caftaric Acid	C_13_H_12_O_9_	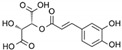	[15,19,56,59,61]
Gentisic Acid	C_7_H_6_O_4_	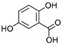	[15,19,56,59,61]
Chlorogenic Acid	C_16_H_18_O_9_	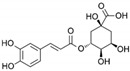	[15,19,56,59,61]
Caffeic Acid	C_9_H_8_O_4_	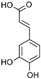	[15,19,56,59,61]
Ferulic Acid	C_10_H_10_O_4_	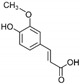	[15,19,56,59,61]
Cinnamic Acid	C_9_H_8_O_2_	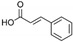	[15,19,56,59,61]
Gallic Acid	C_7_H_6_O_5_	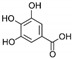	[15,19,56,59,61]
*p*-Coumaric Acid	C_9_H_8_O_3_	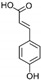	[15,19,56,59,61]
Quercitin	C_15_H_10_O_7_	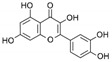	[62,63]
Myricetin	C_15_H_10_O_8_	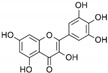	[62,63]
Kaempferol	C_15_H_10_O_6_	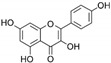	[62,63]
Isorhamnetin	C_16_H_12_O_7_	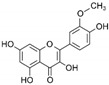	[62,63]
Phloroglucinol	C_6_H_6_O_3_	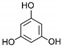	[56,59,62,63]
Pyrocatechol	C_6_H_6_O_2_	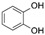	[56,59,62,63]
Pyrogallol	C_6_H_3_(OH)_3_	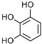	[56,59,62,63]
Vanillin	C_8_H_8_O_3_	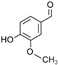	[56,59,62,63]
4-Hydroxybenzaldehyde	C_7_H_6_O_2_	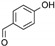	[56,59,62,63]
3,4-Dihydroxybenzaldehyde	C_7_H_6_O_3_	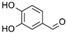	[56,59,62,63]
Benzoic acid	C_6_H_5_COOH	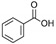	[56,59,62,63]
4-Hydroxybenzoic acid	C_7_H_6_O_3_	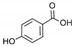	[56,59,62,63]
*p*-Anisic acid	C_8_H_8_O_3_	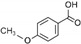	[56,59,62,63]
Vanillic acid	C_8_H_8_O_4_	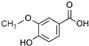	[56,59,62,63]
Syringic acid	C_9_H_10_O_5_	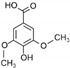	[56,59,62,63]
Phloretin	C_15_H_14_O_5_	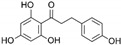	[56,59,62,63]
Phlorizin	C_21_H_24_O_10_	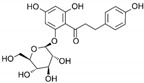	[56,59,62,63]
Palmitic acid	C_16_H_32_O_2_	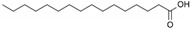	[64]
Palmitoleic acid	C_16_H_30_O_2_	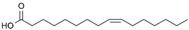	[64]
Oleic acid	C_18_H_34_O_2_	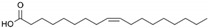	[64]
Linoleic acid	C_18_H_32_O_2_	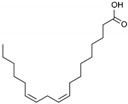	[64]
Campesterol	C_28_H_48_O	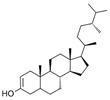	[64]
Stigmasterol	C_29_H_48_O	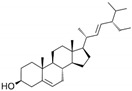	[64]
β-Sitosterol	C_29_H_50_O	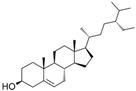	[64]
Posidozinol	C_16_H_32_	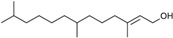	[66]

## Data Availability

Not applicable.
